# Intrarenal venous flow patterns – Guiding fluid management in sepsis with AKI: A case report

**DOI:** 10.1097/MD.0000000000039280

**Published:** 2024-08-09

**Authors:** Qian Zhang, Yi Li, Lixia Liu, Zhenjie Hu, Yan Huo

**Affiliations:** a Department of Intensive Care Unit, The Fourth Hospital of Hebei Medical University, Shijiazhuang, China; b Department of Anesthesiology, Hebei General Hospital, Shijiazhuang, China.

**Keywords:** acute kidney injury, intrarenal venous flow patterns, sepsis

## Abstract

**Introduction:**

Sepsis often leads to acute kidney injury (AKI), presenting significant challenges in fluid management. This study explores the potential of analyzing intrarenal venous flow (IRVF) patterns to guide tailored fluid therapy, aiming to improve patient outcomes.

**Patient concerns:**

A patient was admitted to the intensive care unit with symptoms of septic shock, including fever, severe hypotension, and altered mental status, secondary to a perforated ascending colon adenocarcinoma.

**Diagnosis:**

The patient was diagnosed with perforated ascending colon adenocarcinoma, septic shock, and AKI. Clinical findings included elevated inflammatory markers and impaired renal function.

**Interventions:**

The primary therapeutic interventions included surgical resection of the perforated colon, administration of broad-spectrum antibiotics, and fluid resuscitation. Fluid management was guided by continuous monitoring of IRVF, which facilitated precise adjustments to optimize fluid balance and renal perfusion.

**Outcomes:**

By utilizing IRVF patterns to guide fluid therapy, the patient’s circulatory status and renal function significantly improved. The individualized fluid management approach contributed to better stabilization of the patient’s condition.

**Lessons:**

This case underscores the potential utility of IRVF patterns in guiding fluid management strategies for patients with sepsis and AKI. The main is the benefit of IRVF-guided fluid therapy in improving patient outcomes. Further research is warranted to validate the efficacy and safety of this approach, with the aim of enhancing clinical outcomes in critically ill patients.

## 1. Introduction

Fluid overload is common during sepsis resuscitation and is associated with an increased incidence of acute kidney injury (AKI).^[1–3]^ AKI is a severe complication that significantly impacts patient outcomes. In recent years, the intrarenal venous flow (IRVF) patterns has garnered growing attention for its potential role in the fluid management of critically ill patients with AKI. Renal congestion, characterized by elevated intrarenal interstitial pressure and impaired intrarenal venous return, is a proposed mechanism underlying AKI following fluid overload. IRVF patterns can serve as a crucial indicator for assessing renal filling status and function, thereby playing a pivotal role in individualized fluid management strategies.

Several studies have explored the application of IRVF patterns in critically ill patients, revealing that changes in IRVF patterns closely correlate with alterations in renal function. Particularly in cases of septic shock, abnormal IRVF patterns may signal renal congestion and the onset of AKI. Utilizing IRVF patterns to guide fluid management has demonstrated potential benefits, underscoring its relevance in managing critically ill patients with AKI.^[4–6]^ As ongoing research and clinical validation of this technique progress, IRVF patterns is poised to become an essential tool in future individualized fluid management strategies, offering more effective support for enhancing patient outcomes.

This case emphasizes the potential role of IRVF patterns in fluid management and aims to provide preliminary insights into its application in the fluid management of patients with sepsis, laying the foundation for further research and clinical practice to improve the prognosis of such patients.

## 2. Case presentation

A 57-years-old woman (height 175 cm, weight 70 kg). The patient presented to the local hospital 10 days ago with right lower abdominal pain, which was intermittent dull pain, occasionally accompanied by hematochezia. Colonoscopy revealed a protruding neoplasm near the cecum, and the pathological diagnosis was adenocarcinoma. Abdominal enhanced CT suggested a malignant lesion in the lower part of the ascending colon, with invasion of the adjacent psoas major muscle; acute appendicitis with effusion and surrounding inflammatory changes were also observed. The patient received hydration, anti-inflammatory, and transfusion therapy at the local hospital, but symptoms did not improve significantly. Subsequently, he was transferred to our hospital and diagnosed with “ascending colon adenocarcinoma,” and then underwent surgical treatment.

*Surgical treatment process.* The patient underwent radical right hemicolectomy, including distal closure and proximal diversion, and underwent incision and drainage of the retroperitoneal abscess. During the surgery, a tumor was found in the cecum with a perforation of about 3 mm on the posterior wall, forming a retroperitoneal abscess extending along the dorsal, spinal, and caudal sides of the iliopsoas muscle. Considering the complexity of the condition, the orthopedic consultation suggested temporarily not performing incision and drainage of the right lower limb to control the infection. During the surgery, the patient was hemodynamically unstable and oliguric, and was urgently transferred to the intensive care unit (ICU). Upon admission to the ICU, the patient’s temperature was 36.6 °C, heart rate was 80 beats/min, respiratory rate was 19 breaths/min, blood pressure was 112/72 mm Hg (norepinephrine (NE) 0.7 µg/kg/min), and oxygen saturation was 100%. The patient was in a state of anesthesia, with endotracheal intubation and assisted ventilation with a ventilator, and the abdomen was externally fixed with an abdominal band, with placement of drainage tubes (retroperitoneal), pelvic drainage tube, and abdominal drainage tube (right lower limb), and an ileal stoma was created. The right lower limb was swollen, with no other abnormalities on physical examination. Past medical history: The patient had a history of diabetes for 2 years, with a maximum blood glucose level of 12 mmol/L. He was taking metformin and glimepiride orally, but the specific doses were unknown. He denied a history of hypertension, coronary heart disease, hepatitis, tuberculosis, and other infectious diseases. Laboratory tests showed a white blood cell count of 3.49 × 10^9/L, neutrophil count of 9.29 × 10^9/L (elevated), and platelet count of 71.0 × 10^9/L (decreased). Biochemical examination showed a procalcitonin (PCT) level of 11.55 ng/mL, C-reactive protein (CRP) level of 90.43 mg/L, and creatinine (Scr) level of 103 µmol/L (baseline Scr level: 72 mmol/L). Microbiological examination of drainage fluid revealed *Streptococcus viridans*. Blood gas analysis (upon admission to the ICU): PH 7.323, PCO2: 35 mm Hg, PO2: 90 mm Hg, Lac 5.7 mmol/L. Diagnosis upon admission to the ICU: perforated ascending colon adenocarcinoma; retroperitoneal and right lower limb infection; septic shock; AKI; acute appendicitis.

**Figure 1.**
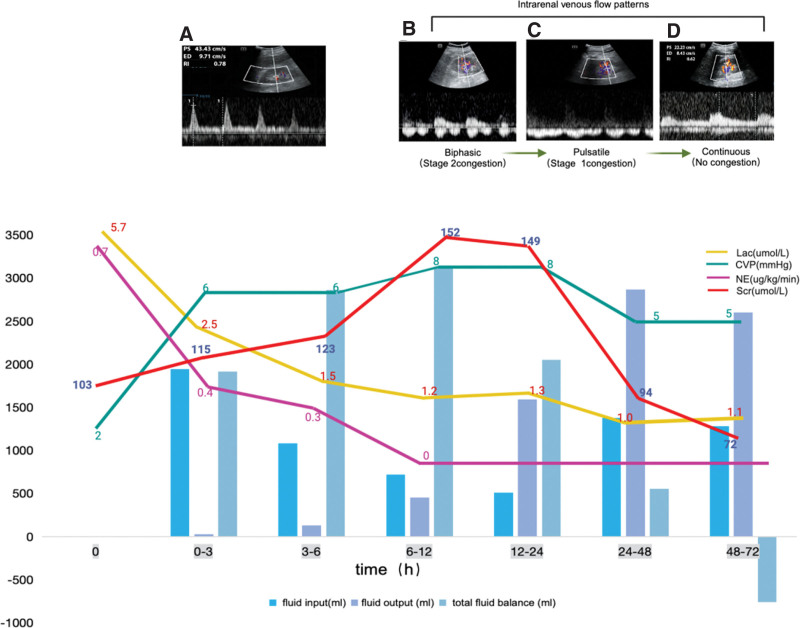
Intrarenal venous flow (IRVF) pattern-guided fluid management. This figure depicts the dynamic changes in various clinical parameters (including lactate levels, central venous pressure, vasopressor usage, creatinine levels, and fluid volume) over the patient’s ICU stay, guided by IRVF patterns. (A) Renal artery resistance index upon ICU admission. (B) At 12 h post-ICU admission, IRVF pattern indicating Stage 1 congestion: pulsatile venous flow. (C) IRVF pattern indicating Stage 2 congestion: biphasic venous flow. (D) No congestion indicated by continuous venous flow. These patterns reflect the progression from renal congestion to a more stable renal hemodynamic state.

*Treatment course after admission to the ICU.* Intravenous antibiotics were administered to control infection, and hemodynamics were stabilized. Dynamic assessment of cardiac, pulmonary, and renal ultrasound, and monitoring of blood lactate (Lac) and intra-abdominal pressure levels were performed. Bedside ultrasound examination upon admission to the ICU showed that the heart was in a high-power state, with inferior vena cava diameter <1 cm, variation of 25%, CVP2 mm Hg, and Lac 5.7 mol/L, suggesting possible hypovolemia. Bilateral renal blood flow was grade 1, with a renal resistance index of about 0.78 (Fig. 1A), and intra-abdominal pressure was 7 mm Hg. After the above treatment, the fluid balance within the first 3 hours was 1915 mL, and the dose of NE was reduced from 0.7 to 0.4 µg/kg/min. Lac decreased by 2.5 mol/L. CVP level increased from 2 to 6 mm Hg, and urine output returned to normal (about 40 mL/h), but Scr increased to 115 µmol/L. The patient’s fluid responsiveness was evaluated as positive, and fluid balance was continued to maintain a positive balance. Subsequent dynamic assessment of the patient’s volume status and timely adjustment of fluid therapy strategies were performed. From the 3rd to the 12th hour, fluid balance was 1221 mL, NE was gradually reduced until discontinued, Lac decreased to 1.2 mol/L, CVP level increased to 8 mm Hg, urine output was normal, but Scr continued to increase to 152 µmol/L, at which point the patient’s fluid responsiveness was evaluated as negative.

*Renal blood flow adjustment strategy.* Further assessment of renal blood flow showed grade 3, and IRVF showed a biphasic discontinuous pattern (Fig. 1B), indicating a large retrograde load on the kidneys and the presence of renal congestion. While ensuring adequate perfusion, a fluid-negative balance therapy strategy was implemented based on the IRVF findings. During the treatment process, Scr levels and IRVF patterns (Fig. 1C) were monitored until IRVF patterns became continuous, and Scr levels gradually returned to baseline. From the 12th to the 48th hour, fluid balance was negative 3896 mL, Lac was 1.1 mol/L, CVP decreased to 5 mm Hg, Scr decreased to 72 mmol/L, and the evaluated renal blood flow was grade 3, with a renal resistance index of 0.62, and renal IRVF showed a continuous pattern (Fig. 1D), and later optimization of hemodynamics was continued.

Outcomes: By utilizing IRVF patterns to guide fluid therapy, the patient’s circulatory status and renal function significantly improved. Initially, the patient presented with high Lac levels (5.7 mmol/L), low central venous pressure (CVP, 2 mm Hg), and impaired renal function (Scr 103 µmol/L). Following IRVF-guided fluid management, Lac levels decreased to 1.1 mmol/L, CVP increased to 6 to 8 mm Hg, and urine output normalized. Scr levels initially rose to 152 µmol/L but gradually returned to baseline (72 µmol/L) as renal perfusion improved. The patient’s hemodynamic status stabilized, and vasopressor support was successfully weaned off. The patient was transferred to a general ward after 5 days of ICU treatment, highlighting the effectiveness of the individualized fluid management approach.

## 3. Discussion

This study delves into the intricate details surrounding the management of a critically ill patient grappling with sepsis compounded by AKI, shedding light on the potential utility of IRVF patterns analysis in fluid management. The case involves a patient admitted to the ICU following a diagnosis of perforated ascending colon adenocarcinoma, complicated further by septic shock and AKI. The renal impairment in such cases could be multifactorial, stemming from factors like septic shock-induced hypoperfusion, hemodynamic fluctuations during surgical interventions, and challenges in volume management amid fluid resuscitation efforts.^[7]^ Notably, analysis of IRVF patterns uncovered renal congestion, a probable contributing factor to the development of AKI.^[8]^

During the treatment and recovery process, CVP levels remained within a relatively normal range, NE dosage gradually decreased until discontinued, urine output remained normal, and systemic circulation gradually stabilized, but renal function worsened. According to the renal ultrasound evaluation, as fluid-negative balance therapy was implemented, IRVF patterns gradually became continuous, and Scr levels gradually returned to baseline. This indicates that adjusting fluid balance strategies has had a positive effect, successfully optimizing the patient’s circulatory status and renal function. This emphasizes the importance of individualized fluid management strategies in critically ill patients.

This case uses IRVF patterns as a guiding tool for fluid management appears to be relatively novel. Traditional fluid management strategies often result in variable outcomes, with some studies highlighting the critical role of meticulous fluid management in improving outcomes for AKI patients. For example, while standard fluid management practices have shown variable renal recovery and a higher risk of fluid overload, the integration of Doppler-guided fluid therapy in heart failure. Patients has led to enhanced renal perfusion and reduced AKI incidence.^[9,10]^ Similarly, conservative fluid management in septic shock patients has improved hemodynamic stability but yielded inconsistent renal outcomes.^[11]^ The current case, however, demonstrates that IRVF-guided fluid management can lead to improved renal function and stable systemic circulation, suggesting a more precise and effective approach to managing fluid balance in critically ill patients.

In summary, this study serves as a testament to the pivotal role of IRVF patterns analysis in informing fluid management strategies for critically ill patients, offering a glimpse into the promising vistas of personalized therapeutic interventions tailored to the unique physiological milieu of individual patients. By harnessing the power of IRVF monitoring, clinicians can navigate fluid resuscitation endeavors with heightened precision, forestalling the perils of fluid overload and mitigating the progression of renal injury. As the quest for optimized patient care continues, the integration of IRVF monitoring into routine clinical practice holds immense potential for revolutionizing the management paradigms governing sepsis complicated by AKI, thereby bolstering the prospects for improved patient outcomes and enhanced quality of care.

This study also has limitations. It is based on a single case report, which limits the generalizability of the findings. The small sample size and the lack of a control group make it difficult to draw definitive conclusions. Additionally, the reliance on IRVF patterns as the sole guide for fluid management may not capture all the complexities of sepsis-induced AKI. Further research involving larger sample sizes and controlled trials is necessary to validate the efficacy and safety of IRVF-guided fluid therapy in diverse clinical settings.

Despite the favorable treatment outcomes observed in this case, vigilance regarding potential complications remains paramount, particularly concerning the risk of hypovolemia accompanying negative fluid balance therapy. Hence, diligent monitoring of patient hemodynamic status during fluid balance adjustments assumes critical importance, aimed at averting the peril of inadequate organ perfusion stemming from hypovolemic states.

This comprehensive exploration underscores the transformative potential of renal Doppler ultrasound technology in the realm of fluid management, charting a course toward enhanced clinical efficacy. Through the implementation of IRVF-guided individualized fluid management protocols, tangible improvements in both circulatory status and renal function were realized, marking a significant stride in the care of critically ill patients with sepsis and AKI.

## 4. Conclusion

This case provides practical experience in the application of IRVF patterns in fluid management of patients with acute sepsis complicated by AKI. Monitoring changes in IRVF during fluid adjustment helps assess renal status more accurately and provides a scientific basis for individualized fluid management strategies. Further research is needed to validate the efficacy and safety of this method and promote its widespread application in clinical practice.

## Acknowledgments

We are grateful to the patient and all the researchers, including the physicians, nurses, and technicians, who participated in this study.

## Author contributions

**Investigation:** Qian Zhang.

**Writing – original draft:** Qian Zhang.

**Resources:** Yi Li.

**Writing – review & editing:** Lixia Liu, Zhenjie Hu, Yan Huo.
